# Vaccination Communicated from the Mother to the Fœtus in Utero

**Published:** 1830-01-01

**Authors:** 


### XXXII.

### Vaccination communicated from the Mother to the Fœtus in Utero.

If the following case is a fact it is curious, if it is not we cannot help it. It comes as well authenticated as many other statements, which pass current for good coin in the scientific course of exchange. It is extracted from the *Kongl. Vetenskaps Academiens Handlingar de* 1817, by the editors of the Journal de Progrés, from which we take it at second hand. It seems that the members of the Academy of Sciences at Stockholm were informed that a woman, who had been vaccinated nine days before her confinement, had given birth to a child which in a few days presented the vaccine vesicle. The learned body in question immediately took steps to investigate into the accuracy of the statement and addressed the requisite enquiries to a clergyman, by name Sædenius, residing at Degerfoss near Umea, amongst whose parishioners were the parents of the child. The clergyman immediately repaired to the spot in company with the sacristan who had vaccinated the woman, and after having taken depositions from the parents and other persons in the same house, he transmitted the following report to the Academy.

"The peasant Maja Brita Andersdotter, living at Oefver-Koede By, aged twenty one years and pregnant for the first time, was vaccinated on the 3d of April, 1813, by my sacristan Rheu, the vaccinator of the parish. On the 12th of the same month the vesicles were so well formed that Rheu took some vaccine from them at 8, a. m. At 4, p. m. the woman Andersdotter was brought to bed of a healthy female infant who was shortly afterwards carried to the baptismal fount, two leagues from the parent's house. The child was brought up by hand with fresh cow's milk and throve well, but at this time it was perceived to have upon its arms the regular vaccine tubercles, in the same number and at the same points as those which had previously appeared upon the mother. The tubercles went on to maturation, left cicatrices after them, and agreed in every respect with those upon the mother. The child went on well for about six weeks, when it died after three days illness from an accidental affection of the chest and stomach."

Many remarks and more speculations might be made upon the foregoing statement, supposing it accurate in every respect. We prefer, however, giving it to our readers as we find it.

---

